# Appraising eHealth Investment for Africa: Scoping Review and Development of a Framework

**DOI:** 10.3390/ijerph21101277

**Published:** 2024-09-25

**Authors:** Sean C. Broomhead, Maurice Mars, Richard E. Scott

**Affiliations:** 1 Department of TeleHealth, School of Nursing & Public Health, College of Health Sciences, University of KwaZulu-Natal, Durban 4041, South Africa; 2 Health Information Systems Program South Africa, Pretoria 0181, South Africa; 3 African Centre for eHealth Excellence, Cape Town 7130, South Africa; 4 Department of Community Health Sciences, Cumming School of Medicine, University of Calgary, Calgary, AB T2N 4Z5, Canada

**Keywords:** eHealth, digital health, investment appraisal, economic appraisal, framework, Africa

## Abstract

Background: As opportunities grow for resource-constrained countries to use eHealth (digital health) to strengthen health systems, a dilemma arises. Wise eHealth investments require adequate appraisal to address opportunity costs. Economic appraisal techniques conventionally utilised for this purpose require sufficient economic expertise and adequate data that are frequently in short supply in low- and middle-income countries. This paper aims to identify, and, if required, develop, a suitable framework for performing eHealth investment appraisals in settings of limited economic expertise and data. Methods: Four progressive steps were followed: (1) identify required framework attributes from published checklists; (2) select, review, and chart relevant frameworks using a scoping review; (3) analyse the frameworks using deductive and inductive iterations; and, if necessary, (4) develop a new framework using findings from the first three steps. Results: Twenty-four candidate investment appraisal attributes were identified and seven relevant frameworks were selected for review. Analysis of these frameworks led to the refinement of the candidate attributes to 23 final attributes, and each framework was compared against them. No individual framework adequately addressed sufficient attributes. A new framework was developed that addressed all 23 final attributes. Conclusions: A new evidence-based investment appraisal framework has been developed that provides a practical, business case focus for use in resource-constrained African settings.

## 1. Introduction

### 1.1. Obstacles to eHealth’s Role in Improving Universal Health Coverage in Africa

The World Health Organization (WHO) defines ‘eHealth’ as “the use of information and communication technologies ICT for health” [1]. There is growing recognition of its role in attaining Universal Health Coverage (UHC) in Africa, where eHealth has many uses, such as improving the safety and quality of clinical decisions by making relevant data available to clinicians, providing health workers with access to knowledge and learning, or using telehealth applications helping to improve people’s access to health services in hard-to-reach areas [2].

Nevertheless, in Africa, people are poor and connectivity, an important requirement for most eHealth, is expensive [3,4]. Under these conditions, eHealth (encompassed by digital health) requires adequate justification to secure investment and achieve its potential to improve UHC [5,6,7,8]. For example, in sub-Saharan Africa, 40% of people live on less than USD 1.90 a day [3,9], and the cost of connectivity, relative to per capita income, is higher than in any other region [10]. There is a strong correlation (0.87 globally) between internet access and a country’s wealth [11], with poorer countries experiencing inadequate connectivity due to the “significant affordability gap” [4]. This is part of a broader digital divide of “economic, educational, and social inequalities between those who have computers and online access and those who do not” [12]. A suitable investment appraisal approach is needed to address the opportunity costs associated with eHealth investments in these resource-constrained settings.

The growth of digital economies was heightened during the COVID-19 pandemic [13,14] and more opportunities are emerging for eHealth [15]. Despite this, and after more than a decade of encouragement by the WHO, World Bank, and others for countries to invest in eHealth [16,17,18], resource-constrained African countries continue to experience significant barriers in the use of eHealth to attain UHC [2,19].

### 1.2. The Role of eHealth Investment Appraisal

To increase investment in eHealth, opportunity costs need to be appraised to identify those eHealth initiatives that offer local stakeholders optimal and affordable net benefits over time [20], when compared with other similar investments. The stakeholders include health system policy and decision makers at various levels in the health system, including hospital and district health managers, who are faced with the task of making decisions about which eHealth initiatives to implement. ‘Investment appraisal’ is the process by which this should be clarified, assessing the relative value of investment options, and providing the rationale and justification for an investment choice.

Formulating this as a definition, ‘eHealth investment appraisal’ is the “process to evaluate which information and communication technology investment in health produces optimal net benefits” [21]. It combines the definition of *investment*, “the act of putting money, effort, time, etc. into something to make a profit or get an advantage” [22], with *appraisal*, “the act of examining something … in order to judge [its] qualities, success, or needs” [23], and applies these to *eHealth* [1]. These principles remain relevant, whether the term is eHealth or digital health and despite their varying and contrasting definitions [24,25]. For the purposes of this paper, they are considered to be synonymous.

Investment appraisal reduces the risk of dysfunctional resource allocations, which can have a direct impact on patient lives and broader society [26]. It does this by using economics principles to assess the costs and benefits of an eHealth initiative over time by providing a framework for making optimal decisions, thereby guiding different stakeholders who may initially disagree. Nevertheless, even in higher-income countries, there are reports of reactionary expenditure on innovations responding to COVID-19 and “emotional accountability” by governments to avoid questions about longer-term sustainability [27]. There has been a call for reform of accounting practices in healthcare [28] supported by new frames of social and moral accountability [26,27,29]. There is recognition of a conflict between accounting and economics approaches in the healthcare setting, with recent calls for changes in accounting practices to support value-based healthcare strategies and the role of digital health investment in achieving them [26].

These dynamics build on half a century of investment appraisal evolution aimed at addressing the shortcomings of pure economic methods, such as dealing with affordability and risk [30] and strengthening the sophistication of appraisal techniques [31]. These issues are pertinent for African countries, where affordability and risk are critical elements.

eHealth investment appraisal must evaluate which information and communication technology investment in health produces optimal net benefits [21]. It should consider outcomes across the health and social contexts in which eHealth is implemented, acknowledging the different stakeholders it is intended to benefit, each of which may have a different perspective on value and benefits [32,33].

### 1.3. Examining eHealth Investment Appraisal Frameworks

There are several ‘health’ economic impact assessment approaches described in the literature [34,35,36]. Few are specific to ‘eHealth’, of which three examples have been highlighted [20]. First, the 2008 European eHealth IMPACT study [37] presents an approach that relies primarily on Cost–Benefit Analysis but places insufficient emphasis on aspects essential to African countries, such as affordability and risk. Second, a 2017 stage-based approach [38] describes a process for selecting the types of economic and financial evaluation methods to use for mHealth evaluation, and how to integrate them, but does not provide a framework that addresses broader implementation issues of eHealth, and how to address limitations in expertise and/or data. Third, the Digital Health Impact Framework (DHIF), developed by the Asian Development Bank in 2019 [39], addresses significantly more aspects than most frameworks, but misses key implementation aspects relevant to African countries, such as implementation milestones and change management. The DHIF extends the work of the European eHealth IMPACT study, and is based on the Five Case Model (FCM) decision-making tool recommended by the United Kingdom [40,41,42] and New Zealand [43] governments to promote public spending accountability. Regrettably, there is no guidance on how to use these approaches in resource-constrained settings [44].

A recent review of eHealth investment appraisal methods used in African countries found no suitable framework for use in African settings due to narrow perspectives that did not adequately address aspects that are important to African countries, such as affordability and implementation. The review did, however, identify promising characteristics in the FCM [21]. Each case of the FCM addresses a distinct question about a proposed initiative: the strategic case (is it needed?), economic case (is it value for money?), financial case (is it affordable?), commercial case (is there a viable partnership model?), and management case (is it achievable?) [20,40,41]. The FCM recognises the need for investment appraisal to go beyond just economic assessment to help identify other factors needed for a potentially viable solution to provide optimal value [45]. Internationally recognised metrics for eHealth investment appraisal have been used to demonstrate the applicability of the FCM to African countries [20]; however, the FCM alone is not sufficient to cover all issues.

### 1.4. Moving towards an eHealth Investment Appraisal Framework for Africa

An ‘FCM for Digital Health (FCM-DH) checklist for investment appraisal’ was proposed as the foundation for developing a suitable framework for African settings [21]. This was developed by selecting attributes from two checklists (the FCM Checklist for Assessment of Business Cases [46], and the Joanna Briggs Institute (JBI) critical appraisal checklist for economic evaluations [47]) and combining them [21]. The FCM checklist items address appraisal of “the estimated probable value of an initiative’s options within the complex health system it operates in” and “strengthens the justification for investing in initiatives that do well in the five cases” [20]. The JBI items contribute additional issues required for robust economic evaluations.

The combined, more comprehensive, checklist provides a useful guide to eHealth investment appraisal by including key elements identified as missing from current appraisals in African countries. These include addressing the valuation of costs and benefits, risk adjustment, affordability, implementation issues, sustainable business models, and partnerships [21]. The expectation is not that the entire FCM-DH checklist would always be utilised. Rather, it helps to achieve a compromise between sophistication and practical utility [21] by providing a wider list of items that appraisers should strive to address. This compensates for data and expertise limitations needed to perform one part of the list, the economic appraisal [48], and provides a foundation for further work to develop an eHealth investment appraisal framework appropriate to African needs.

The general absence of adequate appraisal of African eHealth investments [21,49,50,51] suggests that it is not easy in African country settings. Indeed, it has been reported that few African eHealth initiatives undergo a prior assessment of any kind [52,53], let alone economic appraisal [44,54]. The challenge is that conventional economic assessment techniques are not easily applied because of both a severe shortage of experienced analytical expertise to conduct economic assessments and a lack of adequate and/or available economic data [21]. Together, these two obstacles hinder appraisals before they can begin, resulting in failure to apply best practice appraisal methods. Without a change in perspective and approach, investment appraisals are likely to remain uncommon in Africa.

### 1.5. Goal of This Study

To paraphrase Warren Buffet, adjusted for the eHealth context, ‘*to invest wisely in eHealth solutions, what is needed is a sound framework to make analytic, evidence-informed decisions*’ [55]. To be effective, the framework should help officials to select optimal and relevant eHealth investments despite limitations in economic data and/or expertise, while being forward-thinking enough to maintain its utility as these limitations diminish. The aim of this study was to identify a suitable framework for appraising eHealth investments in Africa and, if none was identified, to use the proposed combination of existing checklists to develop a new tool.

## 2. Methods

### 2.1. Approach

Data about eHealth investment appraisal framework requirements and available frameworks were collected and analysed, and when no suitable framework for eHealth investment appraisal in Africa was found, a new framework and a description of its intended application were developed. The positivist paradigm [56,57] established an evidence-based foundation through four progressive steps summarised as follows: first, clarify what is needed in an eHealth investment appraisal framework in Africa by collating the required attributes from published checklists (Step 1—identify required framework attributes); second, determine whether a suitable framework already exists, using a scoping review and charting data to describe any frameworks identified (Step 2—select and review relevant frameworks); third, determine whether the identified frameworks from Step 2 were fit for purpose using a mixed inductive/deductive approach, guided by aspects of the Framework Method [58] (Step 3—analyse the frameworks iteratively). In the fourth step, since no suitable framework was identified in the previous steps, the outputs of the previous three steps were used to develop a new eHealth Investment Appraisal Framework for Africa and describe its practical application (Step 4—develop a new framework). A narrative approach was used for reporting, guided by the Standards for Reporting Qualitative Research [59].

### 2.2. Step 1—Identify Required Framework Attributes

Candidate investment appraisal attributes, against which any potential investment frameworks identified in Step 2 would be evaluated, were extracted from the previously identified JBI and FCM-DH investment appraisal checklists, which had been developed according to eHealth investment appraisal principles regarded as appropriate to African countries [21]. The data extraction was achieved using an iterative and reflective process resulting in attributes that were framed as straightforward and accessible questions. Similar attributes were combined into a single descriptor, and where one descriptor was chosen over another, the most comprehensive descriptor was selected. The identified candidate attributes were each assigned to one of the five cases of the FCM and each attribute was assessed to determine whether economic expertise and/or economic data were prerequisites for its application.

### 2.3. Step 2—Select and Review Relevant Frameworks

A scoping review was conducted to identify relevant eHealth investment appraisal frameworks and determine whether a suitable framework already existed. The principles of Arksey and O’Malley for scoping studies [60] were adopted (specifically the following five stages: (1) identifying the research question, (2) identifying relevant studies, (3) study selection, (4) charting the data, and (5) collating, summarising, and reporting the results) to answer the following research question: does a suitable framework already exist for appraisal of eHealth investments in Africa? (Arksey and O’Malley Stage 1—identifying the research question).

The PubMed, Science Direct, Scopus, Web of Science, Google, and Google Scholar databases were searched for articles published between 1 July 2011 and 30 June 2024. The following search string was used for PubMed: ((“digital Health” OR eHealth OR e-Health) AND investment AND framework). Google and Google Scholar search findings were restricted to the first 100 results of each. This was in line with the observation of Haddaway et al. [61] that “Systematic reviews typically screen the first 50 to 100 search records within Google Scholar”, and was as recommended by Bramer et al. [62] for situations when the number of potential references from traditional database searches is expected to be low. Two authors (SB, MM) conducted the searches and removed duplicates. All authors screened the titles and abstracts to determine inclusion status. The inclusion criteria were as follows: described an eHealth or digital health investment appraisal framework, and published in English. Conference proceedings, editorials, and letters to the editor were excluded. Full texts of the selected resources were retrieved and reviewed by all authors and the final selection was based on consensus.

The investment appraisal frameworks described in each paper, as well as their purpose, structure, scope, and key lessons learnt from their application, were charted by one author (SB) and reviewed by all authors; differing views were resolved through consensus. Lessons learnt were insights presented in or extracted from a selected paper and applied as a recommendation to be taken into consideration in future actions relating to eHealth investment appraisal. Forward- and backward-chaining were not performed.

### 2.4. Step 3—Analyse the Frameworks Iteratively

The investment appraisal frameworks selected in Step 2 were compared by analysing the charted data inductively, particularly the key lessons, and determining how the insight gained might be used to support or expand the list of candidate attributes. A structured, iterative process was used to link the charted data to conclusions. Any insight gained that was not already addressed in the candidate attributes from Step 1 was phrased as a question and added to an extended and final list of proposed attributes. Next, the frameworks were compared deductively against the final attribute list, using a three-point Likert scale (yes, unclear, or no) deemed appropriate for this type of data collection [21].

To answer “yes”, the paper needed to include a description of how the attribute was or could be applied, rather than simply mentioning it. One author (SB) carried out the primary review and analysis. Thereafter, all authors collectively and critically assessed the data. In order to address inconsistencies in the definition of terms and the risk of subjective assessment affecting the reliability of data collection during this step, a reflective and iterative process was used to identify and clarify the content, with discussion among all authors to arrive at a consensus.

Given the importance of this step, considerable time was spent reviewing and re-reviewing each paper and framework, ensuring familiarity with content and refining understanding. The proportion of attributes covered by each framework was determined, with the aim of identifying a suitable framework for eHealth investment appraisal in Africa, defined pragmatically as one that addressed more than three-quarters of the required attributes for each of the five cases. The findings were tabulated.

### 2.5. Step 4—Develop a New Framework

Since no suitable framework was identified in Step 3, a new eHealth Investment Appraisal FrameworkInvestment Appraisal Framework (eHIAF) for Africa was developed by combining findings from Steps 1–3 to construct a pragmatic, consolidated framework. These data were: (i) the name, purpose, structure, and method of execution of each investment appraisal framework; (ii) descriptions of lessons learnt or practical steps recommended in the selected papers; (iii) the final list of appraisal attributes; (iv) whether economic data and/or economic expertise were prerequisites for the use of each attribute; and (v) Likert scale scoring of each appraisal framework against the final attributes.

The framework was constructed incrementally by making pragmatic choices about how to optimally include the outputs of each of the first 3 steps. This employed a corollary of constructivist epistemology, cognitive pragmatism (the theory of knowledge in pragmatic perspective) [63]. Criteria for developing the eHIAF were based on the practical (pragmatic) purpose of simplifying the process of making optimal eHealth investment decisions in African countries. Once formulated, a description of how to apply the investment appraisal tool was compiled.

## 3. Results

### 3.1. Step 1—Identify Required Framework Attributes

Twenty-four candidate investment appraisal attributes were extracted from the JBI and FCM-DH investment appraisal checklists [21]. These were refined to eighteen by consolidating potential duplicates or closely related attributes and seeking a subjective balance between too few to be effective and too many to be cumbersome for each case of the FCM-.

The reduction from 24 to 18 attributes involved combining four questions into two and making four substitutions. Combinations: Two JBI questions were combined to form the question “Are costs and outcomes both measured accurately and valued credibly?” and two FCM-DH questions were combined to form the question “Is risk exposure addressed and is there an adjustment for optimism bias?” Substitutions: (1) JBI question “Is there a well-defined question?” was replaced with the FCM-DH question “Is there a case for change?” which was considered more appropriate for investment appraisal purposes. (2) The JBI question “Is there a comprehensive description of alternatives?” was replaced by the more comprehensive FCM-DH question “Is there an analysis of options?” since the JBI criterion required only a description of alternatives (comparison between an intervention and a comparator) [47] while the FCM-DH checklist criterion was more stringent, requiring analysis of intervention alternatives. (3) The FCM-DH question “Is there a sensitivity analysis?” was replaced by the more comprehensive JBI question “Were sensitivity analyses conducted to investigate uncertainty in estimates of cost or consequences?” (4) The FCM-DH question “Are costs and benefits identified?” was replaced by the JBI question “Are all important and relevant costs and outcomes for each alternative identified?”

The consolidated candidate attributes list comprised eight (44%) from JBI and ten (56%) from FCM-DH (Table 1). Each attribute was subsequently aligned with one of the FCM cases: four strategic (22%), eight economic (44%), one financial (6%), four management (22%), and one commercial (6%). Seven of the eight economic case attributes and the one financial case attribute require both economic expertise and economic data (Table 1).

### 3.2. Step 2—Select and Review Relevant Frameworks

**Figure 1:**
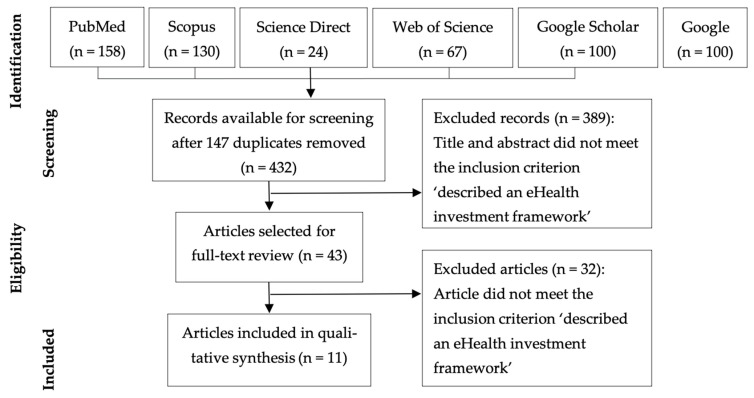
PRISMA flow diagram of search process.

The scoping review identified 432 unique eligible articles (Figure 1; Arksey and O’Malley Stage 2—identifying relevant studies). Screening of abstracts reduced this number to 43, and subsequent full-text review reduced the number that met study inclusion criteria to eleven articles (Table 2). This was further reduced to seven (Table 2, bold; Arksey and O’Malley Stage 3—study selection) based on the following. The authors determined that the framework described by Drury [64] was identical to the one described by Jones [39]. The Drury paper provided reference material, while the Jones paper described the framework. The frameworks described in the Babigumira [65] and Bergmo [66] papers were found to not actually be investment appraisal frameworks. Both restricted their focus to the economic appraisal. The Babigumira report provided a description of how to go about an economic appraisal, along with three case studies. The Bergmo paper provided technical descriptions of several economic appraisal methods and helpful guidance on when each should be used. Both papers contained useful information about how to conduct an economic appraisal though lacked the broader applicability and flexibility of an investment appraisal framework. The quadruple-aim framework described by Woods et al. [67] is included as a component of the Digital Health CBA Framework described by Nguyen et al. [68], and therefore, Woods et al.’s paper was considered redundant and excluded.

Key information about the investment appraisal framework described in each paper was charted (left two columns of Table 3; Arksey and O’Malley Stage 4—charting the data). The lessons from each framework were derived during the extensive and iterative review of each framework. This allowed a comparison of selected frameworks and extraction of lessons for use in the next step (Arksey and O’Malley Stage 5—collating, summarising, and reporting the results).

### 3.3. Step 3—Analyse the Frameworks Iteratively

The charted lessons were analysed to propose how they might support or expand the candidate attribute list (righthand column of Table 3). The impact that each lesson (listed in the middle column) has on the candidate attributes list is recorded immediately alongside that lesson (in the righthand column). Of the twenty-three lessons, eight were already addressed in the candidate investment appraisal attribute list (Table 1), whilst one was not regarded as a relevant addition and was noted for further consideration in the overall design of the new eHealth Investment Appraisal Framework. An additional fourteen contributed to five new attributes addressing stakeholder engagement, connectivity, assumptions and estimates, setting a timescale, and the need for a sustainable business case, all of which are important for successful eHealth.

The twelve lessons addressing stakeholder engagement contributed practical suggestions. For example, the CAF proposed “Having robust features that support clinical use” and that socioeconomic costs and benefits extend across all stakeholder types. The BEF recommended “Evaluating data to establish consensus on ‘best-estimate’ data points” and noted the value of staff engagement and “buy-in”, and the DHIF emphasised that socioeconomic costs and benefits should extend across all stakeholder types. A lesson from the DHCBA was to consider the quadruple aims when exploring value elements. All of these lessons were addressed under the attribute question “Are stakeholders adequately engaged?” and clarified in the instructions about how each should be used (Appendix A—Table A1). The addition of four other new attributes (attributes appended to the word “Add” in the righthand column of Table 3) are clarified by the corresponding lesson alongside each (in the middle column of Table 3) and also included in the instructions (Appendix A—Table A1).

The five additional attributes were each phrased as a question, assessed for whether economic data and/or expertise were required for their use, and allocated to the appropriate FCM case (Table 4).

The addition of these five created a final list of 23 investment appraisal attributes, eight from JBI (35%), ten from FCM-DH (43%), and five from analysis of the seven selected papers (22%) (Table 5). Alignment of the attributes with FCM cases showed five strategic (22%), ten economic (43%), two financial (9%), five management (22%), and one commercial (4%). Seven of the ten economic case attributes, and both the financial case attributes, required economic expertise and data. The possibility of a link between the need for economic expertise and the need for economic data arose during data extraction and analysis. As noted in Table 1 and Table 4, there is no apparent distinction between the need for economic expertise and economic data since, for the attributes identified, some level of both is needed (Table 5).

Likert scores of assessments of the frameworks against the final 23 attributes (Table 1 and Table 4) revealed a binary response of only “yes” and “no”, with no “uncertain” scores (Table 5). None of the five frameworks addressed the required three-quarters of the attributes across all five cases (Table 6). Furthermore, the management case and commercial case were only addressed by one of the frameworks, with neither of the cases having more than half of their attributes addressed.

### 3.4. Step 4—Develop a New Framework

Since none of the five frameworks addressed sufficient attributes across all five cases, a new eHealth investment appraisal framework was developed from the findings of the first three steps. The selected frameworks (Table 3) were compared against the objective of developing an eHIAF for Africa. This revealed advantages, disadvantages, and the contribution of each of the selected frameworks to the development of the new framework (Table 7).

Assessment of the purpose of each framework (Table 7) confirmed that the DHIF aligns best with the requirements for an eHIAF for Africa as revealed by comparison of the proportion of investment appraisal attributes addressed by each of the selected frameworks (Table 6). The DHIF’s ten-step process for business case development is easy to follow, and the DHIF addressed most attributes (18) followed by the BEF (7), BSF (6), CAF (4) and ADF (0). However, since none of the frameworks addressed the required three-quarters of attributes identified, the absence of a suitable framework for eHealth investment appraisal in Africa was confirmed, reinforcing the need for a new framework.

Practical steps incorporated into the new framework were primarily derived from two frameworks (DHIF and BEF), and to a lesser degree from the FEEDH. Five steps of the DHIF ten-step process (identifying stakeholders, benefits, a timescale, conducting economic analysis, and iterating to find optimal relationships) and three of the BEF six-step process (engaging stakeholders in initiation, generating benefit hypotheses, and building a target model) were incorporated. Pertinent lessons gathered from the selected frameworks (such as the importance of simplicity and the need to have a conservative bias) had an impact on how the final attribute listing was organised into six steps (Table 8).

The six stages and twenty-three attributes of the new eHIAF for Africa address all five cases of the FCM. Each stage is associated with one or more attributes to help guide the user through the stage and to be used to evaluate the completeness of an investment appraisal.

The final list of attributes (Appendix A, Table A1) describes how each attribute should be used in the new eHIAF for Africa. These descriptions provide potential users of the six stages of the eHIAF for Africa with a guide presented as checklist items.

## 4. Discussion

### 4.1. Principal Findings

This paper confirms the lack of a suitable eHealth investment appraisal framework for Africa, most noticeable in the sparseness of attributes for the management and commercial cases, and presents the subsequent methodical development of a new framework that provides an evidence-based, practical, business case focus. The framework is appropriate for use in resource-constrained settings that may not have sufficient economic data and/or expertise. The new eHIAF for Africa has six stages, each associated with one or more of 23 specifically selected attributes that, in combination, address all five cases of the FCM to which the framework is aligned (Table 8). The stages are (1) establish a compact with key stakeholders, (2) collect data, (3) generate an economic model, (4) establish affordability metrics, (5) iterate to consider options and identify optimal investment choices, and (6) establish sustainable implementation.

### 4.2. Rationale for Developing a New Framework

There are very few publications describing the use of investment appraisal frameworks for eHealth in Africa [21]. The scoping review in this current paper, which considered frameworks used anywhere in the world, found only seven examples. Examination and analysis showed that none of the seven frameworks addressed more than half of required attributes in every FCM case (Table 5 and Table 6), justifying the need to do things differently and reinforcing the need for a new framework. The attributes of the strategic case were most addressed (23 of 35; 66%) and attributes of the management case the least (1 of 28; 4%). Only one of the five management case attributes (change management) was addressed (and only by the DHIF). Of importance, the other four management case attributes (delivery plan, delivery milestones, procurement plan, connectivity) were not addressed by any of the seven frameworks, reinforcing the need for a new framework. Furthermore, five of the seven papers applied appraisal frameworks in a high-income setting, two in Canada, two in the United States, and one in Australia. One, the FEEDH, had global relevance, and one, the DHIF, is intended for use in low- and middle-income countries (LMICs). The DHIF contributed more to the new framework than the other six frameworks.

More than half of the new framework’s attributes (14 of 23; 61%) can be applied without economic data or expertise. This increases the likelihood of the framework being applied and of an informed decision being made, and also of re-appraisal to support decisions about further investment as new economic data and/or expertise become available.

### 4.3. Appropriateness of the New Framework

Several features of the eHIAF for Africa will help to promote its utilisation. First, the questions are in a simple, easily understandable form. Second, the eHIAF for Africa is presented as a series of stages, each fulfilled through responding to straightforward questions, allowing users to proceed, even in the face of insufficient economic expertise and/or data, and to add to the appraisal as expertise and data improve. Third, by identifying the absence of desirable information, or inability to address certain eHIAF attributes, it will provide perspective and support reflection regarding choice of eHealth intervention, potentially stimulating processes whereby the needed information is made available for future assessments.

In the absence of sufficient economic expertise and/or data, there is a risk that traditional investment appraisal will be limited, or no assessment will be conducted at all. This is the outcome reported from some African countries [52,53]. Therefore, a framework that expands beyond the economic case, such as the eHIAF, increases the likelihood that an investment appraisal that supports decision making will be completed, even if the economic case is imperfect. The expertise needed for the strategic, management, and commercial cases involves an understanding of the health implementation setting and is likely to be more readily available within existing public health resources. Thus, it is possible to partially complete the framework and still have a useful result (such as by omitting the nine attributes that require economic data and/or expertise), although the only way to complete all parts of the framework is to address the limiting factors. This can be accomplished by, for example, outsourcing to bring in economic expertise and/or to specifically gather the required data. As a consequence, to set expectations regarding the framework attributes that can be addressed with available in-house economic expertise and/or data, their extent should be considered first before commencing with an investment appraisal.

One of the attributes added during Step 3 addressed the need for adequate connectivity, a prerequisite for most eHealth and particularly for large-scale implementations [9]. While high-bandwidth connectivity may be ubiquitous in most high-income country settings, in African countries, it can be a significant barrier to some eHealth initiatives. In light of the ongoing and expanding digital divide [74,75], addressing adequate connectivity is an essential component of eHealth investment appraisal in Africa.

The new framework’s underlying FCM structure is well aligned to the six components of the eHealth Economic Evaluation Framework [76], having a perspective, options, timeframe, input costs, outcomes, and method of analysing or comparing options. It also responds to a recommendation of a systematic review of health technology assessment frameworks for eHealth, that eHealth appraisal frameworks include standardisation of assessment outcomes [77].

### 4.4. Appropriateness of the Research Approach

Research must be “based on sound rationale that justifies the use of the chosen methodology” for it to be considered credible [78]. The positivist paradigm plays an important role in public health-related research that informs “service provision and health and social care strategies locally, nationally and globally” [79]. Furthermore, there is growing acceptance of the potential value of utilising mixed quantitative and qualitative methods in research of various paradigms, including the positivist paradigm [56,79,80,81]. Nevertheless, the decision to utilise a mixed approach must be justified [82].

While the prescriptive process utilised in this study was primarily quantitative, significant qualitative work was also required in three of the four steps. The finite list of attributes extracted from published lists in Step 1 was consolidated through iterations of subjective discussions among the authors. Then, during Step 3, iterative discussions were used to extract data from the five selected papers and identify relationships between those data and candidate attributes, to arrive at the final expanded list of attributes. Finally, in the development of the new framework during Step 4, simple listing of the quantitative data extracted from the five selected frameworks would have been insufficient. A qualitative process of iterative subjective discussions was used to arrive at decisions about how the attributes should be addressed in the final framework, including their order and grouping in conceptual phases. Without these qualitative contributions, frameworks would have been assessed against an incomplete list of candidate attributes, and the final framework would have lacked the rigour achieved through the iterative qualitative deliberations.

The principles described by Nowell et al. [83] have been embodied in this study to ensure trustworthiness. They noted that where qualitative research techniques are utilised, “Trustworthiness is one way researchers can persuade themselves and readers that their research findings are worthy of attention”, which depends on credibility, transferability, dependability, and confirmability. This was achieved by describing the steps transparently, making deliberate efforts to thoroughly describe the process and to detail how and why decisions were made so that, within reasonable bounds, other researchers would achieve a similar result.

Cognitive pragmatism provided a pragmatic means to arrive at criteria for developing the eHIAF and helped to ensure a final framework that is practical and appropriate for its purpose [63]. It is an extension of the concept “the map is not the territory” [84]. An illustrative example of cognitive pragmatism applied in a different context is a very simplified map of underground train routes that helps a traveller navigate far more easily than other extremely detailed and more accurate maps; although very simplified, they are appropriate and practical for their purpose [63,85]. Similarly, the eHIAF is appropriate and practical for its purpose, reflecting the essential attributes without unnecessary detail. Peer review of the framework by experts in developed and developing world countries and field testing will be required to validate the framework.

### 4.5. Strengths and Limitations

Strengths relate to the qualitative reporting. The approach has been transparent (based upon available best evidence and first principles), functional (allows flexible application of attributes), straightforward (accessible without profound economic knowledge and/or data), flexible (allowing refinement as availability of economic data and/or expertise improve), and universal (applicable to any eHealth initiative). The implication of this is that it should be repeatable, making it possible for other researchers to examine, criticise and extend the research arguments.

Four limitations have been identified. First, the searches were restricted to English-language literature. Second, the limited number of eHealth investment appraisal frameworks and limited published work describing the frameworks, as confirmed by the scoping review, provided scant proven material to work with. Expanding the number of databases searched, and the search terms, may identify more frameworks. Third, it is also possible that eHealth appraisal is more extensive than reported in published papers, being instead recorded in government documents and eHealth donor reports. Fourth, the intention for the eHIAF for Africa (to be usable without economic expertise and data) may result in weak appraisals that are not able to fulfil the goal of helping to select optimal eHealth initiatives. The implications of these limitations remain speculative until the framework is tested in the field. Expert review and field-testing may provide important additional information to guide further refinements and will provide a more definitive indication of the validity of the framework and the principles used in its development.

### 4.6. Contribution of This Research

Achieving optimal eHealth investment decisions that lead to high-impact African eHealth initiatives requires strengthening at both macro and micro levels. The macro picture had been explored in earlier research that examined the FCM and determined that “its cases are applicable to African countries’ eHealth investment decisions” [20]. Thereafter, a scoping review investigated the micro picture and identified “eHealth investment appraisal approaches and tools that had been used in African countries, described their characteristics and made recommendations regarding African eHealth investment appraisal in the face of limited data and expertise” [21]. This resulted in the suggestion that an extended FCM provided “the foundation of an African eHealth investment appraisal framework” [21] and stimulated the reported research. Now, this paper builds on the earlier research and presents the eHIAF for Africa, which addresses the micro picture. It provides clear guidance for eHealth decision makers in African countries on what needs to be included in a robust investment appraisal, and how to proceed when faced with economic and data expertise limitations.

The eHIAF complements the eHealth Investment Readiness Assessment Tool (eHIRAT), a tool developed to support macro-level strengthening. The eHIRAT is also based on the FCM and provides a profile of strengths and weaknesses in a country’s broader eHealth environment [20]. It comprises ten metrics (nine component metrics and one summary metric that aggregates the nine) that cover critical aspects of eHealth readiness. Visualisations of the metric scores reveal profiles of countries’ relative performance, highlighting areas for strengthening. The eHIAF can help to strengthen African eHealth investment decisions immediately and remain valuable while countries improve the availability of economic data and/or expertise by focusing on weaknesses identified by the eHIRAT.

## 5. Conclusions

The stepwise, qualitative narrative approach described above provides a detailed description and pragmatic approach to the development of an evidence-based framework for eHealth investment appraisal for use in Africa. The principles used in creating the eHIAF for Africa are likely to be broadly applicable (for example, including emerging areas of digital health such as artificial intelligence and big data applications), and other LMICs (that is, those with similar constraints beyond Africa). Application of the eHIAF for Africa will enhance investment appraisal of eHealth by policy and decision makers, making appraisals more accessible regardless of economic data and/or expertise constraints and allowing progress as these constraints lessen. This should promote the selection of optimal eHealth initiatives that help to hasten the achievement of UHC.

## Figures and Tables

**Table 1:** Candidate investment appraisal attributes, alternatives, source checklists, FCM categorisation, and economic expertise or data requirement.

Candidate Investment Appraisal Attributes	Source Checklist	FCM Case	Need for Economic Expertise	Need for Economic Data
1. Is there a strategic fit between the eHealth initiative and the health strategy?	FCM-DH	Strategic	N	N
2. Is there a case for change?	FCM-DH ^a^	Strategic	N	N
3. Does the appraisal include all issues of concern to users?	JBI ^a^	Strategic	N	N
4. Are the results generalisable to the setting of interest?	JBI	Strategic	N	N
5. Are all important and relevant costs and outcomes for each alternative identified?	JBI ^a^	Economic	N	N
6. Are costs and outcomes measured accurately and valued credibly?	JBI	Economic	Y	Y
7. Are costs and outcomes adjusted for differential timing (discount rate)?	JBI	Economic	Y	Y
8. Is there an analysis of options?	FCM-DH ^a^	Economic	Y	Y
9. Is there an incremental analysis of costs and consequences for these options?	JBI	Economic	Y	Y
10. Is the risk exposure addressed and an adjustment made for optimism bias?	FCM-DH	Economic	Y	Y
11. Were sensitivity analyses conducted to investigate uncertainty in estimates of cost or consequences?	JBI ^a^	Economic	Y	Y
12. Has clinical effectiveness been established?	JBI	Economic	Y	Y
13. Is affordability addressed?	FCM-DH	Financial	Y	Y
14. Is there a practical plan for delivery?	FCM-DH	Management	N	N
15. Are clear delivery milestones provided?	FCM-DH	Management	N	N
16. Is change management addressed?	FCM-DH	Management	N	N
17. Is there a plan for building partnerships?	FCM-DH	Commercial	N	N
18. Is there a procurement plan?	FCM-DH	Management	N	N

^a^ Alternatives for each of these five attributes existed in either the JBI or FCM-DH checklists, and the most comprehensive questions were chosen. Legend: FCM-DH = Five Case Model for digital health; JBI = Joanna Briggs Institute. Y = Yes, N = No.

**Table 2:** List of selected papers and the frameworks described.

Author/s, Date	Title	Framework Name	Location
**Lau et al., 2012** [69]	**Impact of electronic medical record on physician practice in office settings: a systematic review**	**Clinical Adoption Framework (CAF)**	**Canada**
**Hagens et al., 2015** [70]	**Valuing national effects of digital health investments: an applied method**	**Benefits Evaluation Framework (BEF)**	**Canada**
**Edmunds et al., 2017** [71]	**An emergent research and policy framework for telehealth**	**Adapted Donabedian Framework (ADF)**	**USA**
**Jones et al., 2018** [39]	**Digital health impact framework user manual**	**Digital Health Impact Framework (DHIF)**	**Asia**
**Nadig et al., 2021** [72]	**Preliminary development of value scorecards as ICU telemedicine evaluation tools**	**Balanced Scorecard Framework (BSF)**	**USA**
**Wilkinson et al., 2023** [73]	**A framework for the economic evaluation of digital health interventions**	**Framework for Economic Evaluation of Digital Health Interventions (FEEDH)**	**Global**
**Nguyen et al., 2024** [68]	**Cashing in: cost-benefit analysis framework for digital hospitals**	**Digital Health Cost–benefit Analysis Framework (DHCBA)**	**Australia**
Bergmo, 2015 [66]	How to measure costs and benefits of eHealth interventions: an overview of methods and frameworks in digital health	(Various economic methods)	N/A
Drury, 2018 [64]	Guidance for investing in digital health	Digital Health Impact Framework (DHIF)	Asia
Babigumira et al., 2021 [65]	Applied economic evaluation of digital health interventions	Expanded Value Framework	Kenya
Woods et al., 2023 [67]	Show me the money: how do we justify spending health care dollars on digital health?	Quadruple-aim Framework	Australia

Note: The seven papers finally selected are shown in bold font; see ‘Step 2—Select and Review Relevant Frameworks’.

**Table 3:** Summary of each framework, key lessons, and actions taken to update the candidate attributes list.

Framework Description: Purpose, Structure, and Scope	Key Lessons and Practical Suggestions from Selected Papers	Impact of Each Key Lesson on the Candidate Attributes List
**Clinical Adoption Framework [69]**		
**Purpose:** A “conceptual model to describe the factors that influence health information system success”. **Structure:** Dimensions at three levels: (1) micro (quality, use and net benefits), (2) meso (people, organisation and implementation factors), and (3) macro (standards, legislation and governance, funding and incentives, and societal, political and economic trends). **Scope:** Systematic review of electronic health records using the dimensions of the Clinical Adaption Framework to categorise impacts identified in the literature.	Emphasised the following in electronic medical record (EMR) design and implementation:	
	a. “Having robust features that support clinical use”;	a. Add “Stakeholder engagement” to include users in identifying features;
	b. “Redesigning EMR-supported work practices for optimal fit”;	b. Add “Stakeholder engagement” to include users in redesign; also part of “Options analysis” to test optimal fit;
	c. “Demonstrating value for money to encourage electronic medical record adoption/use”;	c. Part of “Users concerns”;
	d. “Having realistic expectations on EMR implementation effort”;	d. Add “Stakeholder engagement” to set expectations;
	e. “Engaging patients in the process to improve the overall encounter experience”.	e. Add “Stakeholder engagement” to engage all users; also part of “Users concerns” attribute to set improvement expectations.
**Benefit Evaluation Framework [70]**		
**Purpose:** “To inform policy, encourage effective adoption, and support clinical practice transformation”. **Structure:** A six-step process, (1) initiation, (2) benefit hypotheses, (3) target model, (4) evidence search, (5) benefit modelling, and (6) peer review, with results communicated in a final report. **Scope:** A mixed-methods approach to validate potential eHealth solutions by a panel of external experts described as an “iterative process that takes 1 to 3 months and engages as many as fifty experts”.	a. The teams generating the models must apply a conservative bias when estimating benefits, yet aim to generate a comprehensive estimate;	a. Add “Stakeholder engagement”; also part of “all important and relevant costs and outcomes for each alternative identified”;
	b. Include sensitivity analysis;	b. Part of “Sensitivity analysis”;
	c. Evaluate data to “establish consensus on ‘best-estimate’ data points”.	c. Add “Stakeholder engagement” to establish consensus in analysis of options.
**Adapted Donabedian Framework** [71]		
**Purpose:** A research and policy framework intended to educate policymakers, payers and health systems about the value of telemedicine. **Structure:** Expert panel interviews that explore the value of telemedicine in three components: (1) structure (policy aspects of regulation and payment), (2) process (delivery system such as providers, clinical settings and technologies), and (3) outcomes along the continuum of care (prevention, access, quality and cost savings). **Scope:** A process to develop a draft policy framework to clarify the value of telemedicine through expert interviews that are validated in an experts’ workshop.	Noted the “urgent need for a systematic, policy-relevant framework to integrate regulatory, operational, and clinical factors and to guide future investments in telehealth research and practice”.	No action required
**Digital Health Impact Framework** [39]		
**Purpose:** To guide “people on how to appraise planned digital health investment decisions” and provide a process for assembling the required data and analyses needed for these decisions. **Structure:** Ten key steps: (1) identify timescales; (2) identify stakeholders; (3) generic schedule of estimated benefits without quantification; (4) identify resources needed; (5) estimate socioeconomic benefits’ monetary values; (6) estimate socioeconomic costs; (7) adjust for sensitivity, optimism, and risk; (8) calculate net benefits, the socioeconomic returns; (9) estimate the financial costs and affordability of strategic scenarios, adjusted for sensitivity, optimism bias, and risk; (10) refine and iterate socioeconomic returns and affordability to find an optimal link. **Scope:** Development of business cases that provide decision-making information regarding individual eHealth implementations.	a. Digital health (DH) investment decisions should “identify a mix of DH projects that offers the best, affordable fit” to health strategies;	a. Part of “Strategic fit”;
	b. “Identify options for each DH project to offer the best affordable value for money”;	b. Part of “Options analysis”;
	c. Adequate investment for connectivity;	c. Add “Adequate connectivity”;
	d. Assessing both costs and benefits relies on costing and pricing methodologies;	d. Part of “Costs and outcomes measured accurately and valued credibly”;
	e. Socioeconomic costs and benefits extend across all stakeholder types, such as patients, their carers, families;	e. Add “Stakeholder engagement” to ensure that “all important and relevant costs and outcomes for each alternative” are identified;
	f. Appropriate assumptions and estimates;	f. Add “Assumptions and estimates”;
	g. Large-scale initiatives take a long time to achieve socioeconomic return;	g. Add “Set a timescale”;
	h. Decision makers need a business case for effective investment decisions.	h. Add “Sustainable business case”.
**Balanced Scorecard Framework** [72]		
**Purpose:** A service development guide as well as a performance management tool for use prior to, during, and after adoption. **Structure:** Sequential, mixed-methods design that uses expert interviews to identify domains and components and then uses the result to generate composite, multidimensional value scorecards to appraise investments in Intensive Care Unit telemedicine, based on four domains of effects: (1) organisational (10 components), (2) clinical (5 components), (3) financial (8 components), and (4) strategic (3 components). **Scope:** A point-based scoring system that hospitals can apply prospectively to appraise potential investments in Intensive Care Unit telemedicine.	a. Emphasise staff engagement and “buy-in”;	a. Add “Stakeholder engagement”;
	b. Foster collaboration among service providers;	b. Part of “Partner plan”;
	c. Consider how users perceive the initiative;	c. Add “Stakeholder engagement”; also part of “Users concerns” to capture perceptions;
	d. Consider how the initiative has affected patient care;	d. Part of “Clinical effectiveness”;
	e. Consider whether the initiative is financially sustainable;	e. Add “Sustainable business case”; also part of “Affordability”;
	f. Examine what must change to increase the value derived from the initiative.	f. Part of “Case for change”.
**Digital Health CBA Framework** [68]		
**Purpose:** A “framework to conduct cost–benefit analysis for [electronic medical record] implementation in digital hospitals”. **Structure:** Four components: 1. Cost–Benefit Analysis, where “positive net-benefit indicates that the project is an efficient investment of resources, compared to its alternatives, from an economic point of view”; 2. The quadruple aim for health care, which includes “(1) to improve population health, (2) to enhance the patient experience, (3) to reduce cost per patient, and (4) to improve the work-life balance of healthcare workforce”; 3. The elements of value framework, which promotes a broader view of value in health by adding elements for consideration; 4. The eHealth Evaluation Framework, based on the Clinical Adoption Framework described above in this table. **Scope:** A conceptual framework intended for local adaptation “to calculate the total economic value” of electronic medical record implementations.	Regarding Cost–Benefit Analysis, include:	
	a. Counter-factual scenario;	a. Add the option to *not* implement the proposed digital health initiative in the “Analysis of options”;
	b. Timeframe;	b. Add “Set a timescale”;
	c. Discount rate;	c. Part of “Adjust for differential timing”;
	d. Various stakeholder perspectives including ‘market”, ‘private’, ‘efficiency’, and ‘reference group’;	d. Part of “Users concerns”; add “Stakeholder engagement”;
	e. Quadruple aims when exploring value elements.	e. Part of “Users concerns”; add a clarification that this should include aspects of population health improvements, enhanced patient experience, reduced cost per patient, and improved work–life balance.
**Framework for Economic Evaluation of Digital Health Interventions** [73]		
**Purpose:** To provide a “consistent mechanism for representing the value of [digital health innovations] in context” to “improve decision making and investments in [digital health] under constrained budgets”. **Structure:** Five steps: “(1) determine the context, (2) determine the intervention type, (3) establish the level of complexity, (4) set the analytic principles, and (5) represent the value proposition”. **Scope:** Targets digital health innovations as defined by the WHO classification [8], including further consideration for the evaluation that includes predictive analytics, and other non-digital health interventions enabled by predictive analytics.	Include the following:	
	a. A standardised methodological approach that can be used both prospectively and retrospectively;	a. No action required;
	b. Set a time horizon sufficient to capture anticipated changes;	b. Add “Set a timescale” and clarify the need for it to cover the period to capture anticipated changes;
	c. Comparative analysis of alternative courses of action of both costs and consequences;	c. Part of “Analysis of options”;
	d. Accurately represent costs and consequences in context;	d. Part of “Users concerns” and “incremental analysis of costs and consequences”;
	e. Reflect the complexity of the analytic question;	e. Add “Stakeholder engagement” and add the need to clarify the intervention, causal and outcome complexity with stakeholders, and incorporate these into subsequent steps;
	f. Consider value as multidimensional (clinical, organisational, behavioural, technical);	f. Part of “Users concerns”, adding that clinical, organisational, behavioural, technical value should be considered as multidimensional;
	g. Consider value from a multi-stakeholder perspective;	g. Part of “Users concerns”; add “Stakeholder engagement”;
	h. Represent the risks and uncertainty inherent in digital health analytical methods;	h. Part of “Risk exposure”; add that uncertainty should be included in risk and optimism bias adjustments;
	i. Include an Impact Inventory of health and non-health outcomes and costs.	i. Add an Impact Inventory to the “Change management” attribute.

**Table 4:** Additional candidate attributes identified during data extraction.

Candidate Investment Appraisal Attributes	Source	FCM Case	Need for Economic Expertise	Need for Economic Data
Is there evidence of adequate stakeholder engagement in the appraisal process?	CAF, BEF, DHIF, BSF, DHCBA, FEEDH	Strategic	N	N
Has an appropriate timescale been set?	DHIF, DHCBA, FEEDH	Economic	N	N
Have appropriate assumptions and estimates been established?	DHIF	Economic	N	N
Is there a sustainable business case?	DHIF, BSF	Financial	Y	Y
Is there adequate connectivity?	DHIF	Management	N	N

Legend: FCM = Five Case Model; CAF = Clinical Adoption Framework, BEF = Benefit Evaluation Framework, DHIF = Digital Health Impact Framework, BSF = Balanced Scorecard Framework, DHCBA = Digital Health Cost–Benefit Analysis Framework, FEEDH = Framework for Economic Evaluation of Digital Health Interventions. Y = Yes, N = No.

**Table 5:** Analysis of the presence “Y” or absence “N” of candidate attributes in selected frameworks.

FCM Case	Investment Appraisal Attributes	Framework							Total “Yes”	Economic Expertise and Data Needed
		CAF	BEF	ADF	DHIF	BSF	DHCBA	FEEDH		
Strategic	1. Is there a strategic fit between the eHealth initiative and the health strategy?	N	N	N	Y	Y	N	N	2	N
	2. Is there a case for change?	N	Y	N	Y	Y	Y	Y	5	N
	3. Is there evidence of adequate stakeholder engagement in the appraisal process?	Y	Y	N	Y	Y	Y	Y	6	N
	4. Does the appraisal include all issues of concern to users?	Y	N	N	Y	Y	Y	Y	5	N
	5. Are the results generalisable to the setting of interest?	N	Y	N	Y	Y	Y	Y	5	N
Economic	6. Has an appropriate timescale been set?	N	N	N	Y	N	Y	Y	3	N
	7. Are all important and relevant costs and outcomes for each alternative identified?	Y	Y	N	Y	N	Y	Y	5	N
	8. Have appropriate assumptions and estimates been established?	N	Y	N	Y	N	Y	Y	4	N
	9. Are costs and outcomes measured accurately and valued credibly?	N	Y	N	Y	N	N	Y	3	Y
	10. Are costs and outcomes adjusted for differential timing (discount rate)?	N	N	N	Y	N	Y	Y	3	Y
	11. Is there an analysis of options?	N	Y	N	Y	N	Y	Y	4	Y
	12. Is there an incremental analysis of costs and consequences for these options?	N	N	N	Y	N	N	Y	2	Y
	13. Is the risk exposure addressed and an adjustment made for optimism bias?	N	N	N	Y	N	N	Y	2	Y
	14. Were sensitivity analyses conducted to investigate uncertainty in estimates of cost or consequences?	N	N	N	Y	N	Y	Y	3	Y
	15. Has clinical effectiveness been established?	Y	N	N	N	Y	N	N	2	Y
Financial	16. Is affordability addressed?	N	N	N	Y	N	N	N	1	Y
	17. Is there a sustainable business case?	N	N	N	Y	N	N	N	1	Y
Management	18. Is there a practical plan for delivery?	N	N	N	N	N	N	N	0	N
	19. Are clear delivery milestones provided?	N	N	N	N	N	N	N	0	N
	20. Is change management addressed?	N	N	N	Y	N	N	N	1	N
	21. Is there adequate connectivity?	N	N	N	N	N	N	N	0	N
Commercial	22. Is there a plan for building partnerships?	N	N	N	Y	N	N	N	1	N
	23. Is there a procurement plan?	N	N	N	N	N	N	N	0	N
Total “Yes”		4	7	0	18	6	10	13		9

Legend: CAF = Clinical Adoption Framework, BEF = Benefits Evaluation Framework, ADF = Adapted Donabedian Framework, DHIF = Digital Health Impact Framework, BSF = Balanced Scorecard Framework, DHCBA = Digital Health Cost–Benefit Analysis Framework, FEEDH = Framework for Economic Evaluation of Digital Health Interventions. Y = Yes, N = No.

**Table 6:** Proportion of investment appraisal attributes addressed by each of the selected frameworks by FCM category.

FCM Case	Proportion of Investment Appraisal Attributes Addressed by Each Framework						
	CAF	BEF	ADF	DHIF	BSF	DHCBA	FEEDH
1. Strategic	40%	60%	0%	**100%**	**100%**	**80%**	**80%**
2. Economic	20%	40%	0%	**90%**	10%	60%	**90%**
3. Financial	0%	0%	0%	**100%**	0%	0%	0%
4. Management	0%	0%	0%	25%	0%	0%	0%
5. Commercial	0%	0%	0%	50%	0%	0%	0%

Legend: CAF = Clinical Adoption Framework, BEF = Benefit Evaluation Framework, ADF = Adapted Donabedian Framework, DHIF = Digital Health Impact Framework, BSF = Balanced Scorecard Framework, DHCBA = Digital Health Cost–Benefit Analysis Framework, FEEDH = Framework for Economic Evaluation of Digital Health Interventions. Percentage values are shown in bold font where a framework addressed more than three-quarters of the attributes of an FCM case.

**Table 7:** Summary of each selected framework’s contributions to the new eHealth investment framework for Africa.

Name	Advantage	Disadvantage	Contributions to New Framework
CAF	Simple structure, with extensive list of factors that influence success, arranged according to dimensions	There is no explanation of how to use the components; inadequate coverage of strategic and FCM economic cases and no content for financial, management and commercial cases	Addition of “Stakeholder engagement”; confirmation of “Options analysis” and “Users concerns”
BEF	Thorough process; partial coverage of strategic and economic cases	A long, resource-intensive process intended to inform policy, not assist with the appraisal of individual digital health initiatives; no content for financial, management and commercial cases	Addition of “Stakeholder engagement”; confirmation of “Sensitivity analysis”. Contribution of 3 of the 6 steps of the BEF
ADF	Consultative process	The approach is intended to educate about the value of telemedicine and does not assist with the appraisal of individual digital health initiatives Missing the attributes of all FCM cases	None
DHIF	Has broad applicability and guidance for developing digital health business cases with good coverage of FCM strategic, economic, and commercial cases	The clinical effectiveness attribute is missing from the economic case and four attributes are missing from the management case	Addition of “Stakeholder engagement”, “Connectivity”, “Assumptions and estimates”, “Set a timescale”, and “Sustainable business case”; confirmation of “Strategic fit”, “Options analysis” and “Costs and outcomes measured accurately and valued credibly”. Contribution of 5 of the 10 steps of the DHIF
BSF	The highly flexible approach is applied to ICU telemedicine as an example	No coverage of economic case (except for clinical effectiveness), no content for financial, management and commercial cases, and only modest coverage of the strategic case	Addition of “Stakeholder engagement” and “Sustainable business case”; confirmation of “Partner plan”, “User concerns”, “Clinical effectiveness”, “Sustainable business case”, “Affordability”, and “Case for change”
DHCBA	Expands the economic appraisal for a broader view of value in health when considering the total economic value of medical record implementations	Missing content for financial, management, and commercial cases	Addition of the option to *not* implement the proposed digital health initiative in the “analysis of options”, “Set a timescale”, “Stakeholder engagement”, and clarification that “Users concerns” should include aspects of population health improvements, enhanced patient experience, reduced cost per patient, and improved work–life balance; confirmation of “Adjust for differential timing”, “Users concerns”, and “All important and relevant costs and outcomes”
FEEDH	A flexible approach for representing the value of digital health innovations with specific consideration given to how to approach varying levels of digital health innovation complexity, including specific consideration to predictive analytics, and other non-digital health interventions enabled by predictive analytics	Missing content for financial, management and commercial cases	Addition of “Set a timescale” clarifying the need for it to cover the period of anticipated changes, “Stakeholder engagement” clarifying the intervention, causal and outcome complexity and incorporating these into subsequent steps, adding that user value should be considered as multidimensional, that uncertainty should be included in risk and optimism bias adjustments, and adding an Impact Inventory to the “Change management” attribute; confirmation of “Analysis of options”, “Users concerns”, “Incremental analysis of costs and consequences”, and “Risk exposure”

Legend: CAF = Clinical Adoption Framework, BEF = Benefit Evaluation Framework, ADF = Adapted Donabedian Framework, DHIF = Digital Health Impact Framework, BSF = Balanced Scorecard Framework, DHCBA = Digital Health Cost–Benefit Analysis Framework, FEEDH = Framework for Economic Evaluation of Digital Health Interventions.

**Table 8:** Emerging structure of the new eHealth Investment Appraisal Framework for Africa.

FCM Case	eHIAF Stages	eHIAF Appraisal Attributes ^a^	DHIF ^b^	BEF	FEEDH
Strategic	1. Establish a compact with key stakeholders	1. Is there a strategic fit between the eHealth initiative and the health strategy?	2. Identify stakeholders	Engage stakeholders in initiation	1. Determine the context 2. Determine the intervention type 3. Establish the level of complexity
		2. Is there a case for change?			
		3. Is there evidence of adequate stakeholder engagement in the appraisal process?			
		4. Does the appraisal include all issues of concern to users?			
		5. Are the results generalisable to the setting of interest?			
Economic	2. Collect data	6. Has an appropriate timescale been set?	1. Identify timescales	Generating benefit hypotheses	4. Set the analytic principles
		7. Are all important and relevant costs and outcomes for each alternative identified?	3. Identify benefits 4. Identify resources needed 5. Estimate socioeconomic benefits’ monetary values 6. Estimate socioeconomic costs		
		8. Have appropriate assumptions and estimates been established?			
		9. Are costs and outcomes measured accurately and valued credibly?			
		10. Are costs and outcomes adjusted for differential timing (discount rate)?			
	3. Generate economic model	11. Is there an analysis of options?	7. Adjust for sensitivity, optimism, and risk	Building a target model	5. Represent the value proposition
		12. Is there an incremental analysis of costs and consequences for these options?			
		13. Is the risk exposure addressed and an adjustment made for optimism bias?			
		14. Were sensitivity analyses conducted to investigate uncertainty in estimates of cost or consequences?	8. Calculate net benefits, and socioeconomic returns		
		15. Has clinical effectiveness been established?			
Financial	4. Establish affordability metrics	16. Is affordability addressed?	9. Estimate financial costs and affordability		
	5. Iterate to consider options and identify optimal investment choices	17. Is there a sustainable business case?	10. Refine and iterate socioeconomic returns and affordability to find an optimal link		
Management	6. Establish sustainable implementation	18. Is there a practical plan for delivery?	(No ‘Step’ specifically addresses the management or commercial cases)		
		19. Are clear delivery milestones provided?			
		20. Is change management addressed?			
		21. Is there adequate connectivity?			
Commercial		22. Is there a plan for building partnerships?			
		23. Is there a procurement plan?			

Legend: FMC = Five Case Model, eHIAF = eHealth Investment Appraisal Framework, BEF = Benefit Evaluation Framework, DHIF = Digital Health Impact Framework, FEEDH = Framework for the Economic Evaluation of Digital Health Interventions. ^a^ The numbering of the eHIAF appraisal attributes indicates the order of the proposed new eHIAF steps. ^b^ The numbering of DHIF indicates the order of the original DHIF steps.

## Data Availability

The original contributions presented in the study are included in the article. Further inquiries can be directed to the corresponding author.

## Appendix A

**Table A1:** List of attributes and how each should be used in an eHealth investment appraisal.

Attribute	Requirements for Implementing Each Attribute
**Strategic case**	
1. Is there a strategic fit between the eHealth initiative and the health strategy?	Clear alignment exists between the initiative and the health policy context; the objective is clearly stated and reflects the main stakeholders’ perspectives; there is an appropriate mix of initiatives to offer the best fit to health strategies.
2. Is there a case for change?	There is a clear and concise statement of the required service outputs and requirements with explicit, measurable, and time-bound investment objectives.
3. Is there evidence of adequate stakeholder engagement in the appraisal process?	Stakeholders have been engaged to confirm the case for change and strategic context; identify users’ expectations regarding required features; redesign work practices for optimal fit; ensure that all important and relevant costs and outcomes for each alternative are identified; capture user perceptions, achieve “buy-in” and establish consensus when analysing options; and clarify the intervention, causal, and outcome complexity for these to be incorporated into subsequent steps.
4. Does the appraisal address all issues of concern to users?	The initiative addresses all the questions users/decision makers would ask when deciding about whether or not to proceed with the initiative, capturing stakeholders’ perceptions, and addressing their improvement expectations. This should include, where appropriate, aspects of population health improvements, enhanced patient experience, reduced cost per patient, and improved work–life balance for patients and health workers. It should explore what constitutes value for money for the users, with value considered as multidimensional, including clinical, organisational, behavioural, and technical aspects.
5. Are the results generalisable to the setting of interest?	The implementation setting is adequately described and the issue of transferability of findings to other settings with similar characteristics is addressed.
**Economic case**	
6. Has an appropriate timescale been set?	The timescale describes and justifies the entire lifecycle of the initiative from conceptualisation, through approval and implementation, through any reinvestment phases and on to obsolescence and decommissioning.
7. Are all important and relevant costs and outcomes for each alternative identified?	There is a comprehensive list of all relevant cost and outcomes, including a benefit register, sufficient to address the objectives of the study.
8. Have appropriate assumptions and estimates been established?	All assumptions have been recorded and costs and outcomes estimates have been specified with accompanying ranges.
9. Are costs and outcomes measured accurately and valued credibly?	Selected measurement methods are justified and limitations are discussed, recognising that, in economic evaluations, it is often difficult to measure costs and outcomes accurately, and hence this quality criterion may be difficult to achieve. A persuasive argument is provided for the pricing method used to value costs and benefits.
10. Are costs and outcomes adjusted for differential timing (discount rate)?	A discount rate is justified, aligned with the timescale set in Step 6.
11. Is there an analysis of options?	A clear description of the initiative(s) and comparator(s) is provided to identify options to offer best affordable value for money and find optimal fit for the organisation’s business needs. The analysis is reviewed with key stakeholders, including users, including the option to *not* implement the digital health initiative.
12. Is there an incremental analysis of costs and consequences for these options?	A measure is reported that shows the change in costs and outcomes for the initiative and a comparator for a marginal shift in resources from the comparator to the intervention.
13. Is the risk exposure addressed and an adjustment made for optimism bias?	Key risks have been entered into a risk register, analysed and costed, for use in sensitivity testing. Optimism bias has been examined and applied to the initial estimates. Optimism bias adjusted for risk is reflected in the level of certainty shown by the costs’ and outcomes’ value ranges. There is a description of how risks, optimism bias, and the uncertainties inherent in digital health initiatives have been estimated.
14. Were sensitivity analyses conducted to investigate uncertainty in estimates of cost or consequences?	Sensitivity testing results are presented to confirm the robustness of findings, describing how findings vary with changes in key variables such as relative prices, and intervention estimates. Have early estimates of key benefits and key risks been entered into the benefits and risks registers, respectively, and been used in sensitivity analysis?
15. Has clinical effectiveness been established?	The evidence used to derive a clinical effectiveness estimate, the level of this evidence, and how the estimate was derived are recorded. The evidence may be derived from another initiative.
**Financial case**	
16. Is affordability addressed?	The initiative’s potential and the whole life costs (capital and revenue) over the entire life span of the initiative and sources of funding are clearly identified, agreed among stakeholders, and affordable. The costs of monitoring and evaluating and procurement are included. All indicative financial cost ranges, sources, and assumptions have been updated with the best estimates available. The sum of residual optimism bias and residual risk have been revisited as a basis for estimation of the contingent cost liability. Contingent cost liabilities have been addressed, informed by a likelihood valuation.
17. Is there a sustainable business case?	Iterations are performed to refine the model to establish an optimal link between socioeconomic returns and affordability, with the impacts on balance sheets and cash flows of participating organisations over time reviewed and decision makers in agreement with the business case for the lifecycle of the investment.
**Management case**	
18. Is there a practical plan for delivery?	Working arrangements, operational plans, and resources are in place for the successful implementation of the initiative. Arrangements for governance, monitoring and reporting, assurance, and post-evaluation are in place. Contingency plans are in place. Risks are allocated to the organisation best placed to monitor and manage them.
19. Are clear delivery milestones provided?	There are clear milestones for initiative outputs, including key contractual and delivery arrangements (milestones and dates).
20. Is change management addressed?	A plan is in place to support stakeholders, particularly users, through the changes that need to be made to ensure success of the initiative, utilising an Impact Inventory of health and non-health outcomes and costs identified in prior steps to monitor anticipated changes.
21. Is there adequate connectivity?	There is a plan to ensure sufficient connectivity for the initiative’s success that plausibly addresses all unique local setting challenges.
**Commercial case**	
22. Is there a plan for building partnerships?	Due diligence has been undertaken to ensure the capacity, resilience, and capability of the supply side, and confirm that a potential deal can be made. All envisaged deals have been summarised together with details of the service outputs, timescales, risk apportionment, payment mechanisms, and accountancy treatment, including the accounting treatment of underpinning assets. There are key contractual and delivery milestones and dates. The potential for risk transfer among stakeholders has been addressed, including how risk will be tied down in payment arrangements. There is a robust and enforceable commercial contract, with appropriate contractual clauses.
23. Is there a procurement plan?	There is a clear understanding of the procurement approach. There is a draft advertisement for competitive procurement. Contract lengths have been stipulated, together with any required breakpoints. A joint approach has been agreed with any other affected entities and arrangements are in place to manage that. Investment objectives reflect any adjustments made as a result of procurement.
